# JAK2 activation by growth hormone and other cytokines

**DOI:** 10.1042/BJ20141293

**Published:** 2015-02-06

**Authors:** Michael J. Waters, Andrew J. Brooks

**Affiliations:** *Institute for Molecular Bioscience, The University of Queensland Institute, QLD 4072, Australia; †The University of Queensland Diamantina Institute, The University of Queensland, Translational Research Institute, QLD 4072, Australia

**Keywords:** class I cytokine receptors, cytokine receptor signalling, growth hormone, growth hormone receptor, Janus kinase 2 (JAK2), Srk family kinases, CNTF, ciliary neurotropic factor, CRH, cytokine receptor homology, CT-1, cardiotropin-1, ECD, extracellular domain, EPO, erythropoietin, FNIII, fibronectin III-like, GH, growth hormone, GM-CSF, granulocyte-macrophage colony-stimulating factor, JAK, Janus kinase, JM, juxtamembrane, MAb, monoclonal antibody, OSM, oncostatin-M, PK, pseudokinase, TMD, transmembrane domain, TPO, thrombopoietin

## Abstract

Growth hormone (GH) and structurally related cytokines regulate a great number of physiological and pathological processes. They do this by coupling their single transmembrane domain (TMD) receptors to cytoplasmic tyrosine kinases, either as homodimers or heterodimers. Recent studies have revealed that many of these receptors exist as constitutive dimers rather than being dimerized as a consequence of ligand binding, which has necessitated a new paradigm for describing their activation process. In the present study, we describe a model for activation of the tyrosine kinase Janus kinase 2 (JAK2) by the GH receptor homodimer based on biochemical data and molecular dynamics simulations. Binding of the bivalent ligand reorientates and rotates the receptor subunits, resulting in a transition from a form with parallel TMDs to one where the TMDs separate at the point of entry into the cytoplasm. This movement slides the pseudokinase inhibitory domain of one JAK kinase away from the kinase domain of the other JAK within the receptor dimer–JAK complex, allowing the two kinase domains to interact and *trans*-activate. This results in phosphorylation and activation of STATs and other signalling pathways linked to this receptor which then regulate postnatal growth, metabolism and stem cell activation. We believe that this model will apply to most if not all members of the class I cytokine receptor family, and will be useful in the design of small antagonists and agonists of therapeutic value.

## INTRODUCTION

A substantial number of key class I cytokine receptors utilize Janus kinase 2 (JAK2) for signalling [1,2]. The growth hormone (GH) and erythropoietin (EPO) receptors were the first of these to be reported, and later the prolactin, interleukins 3, 5 and 6, granulocyte-macrophage colony-stimulating factor (GM-CSF), interferon-γ (IFNγ), thrombopoietin (TPO) and leptin receptors were found to recruit this ubiquitous tyrosine kinase. The spectrum of physiological processes regulated by JAK2 is therefore vast, ranging from postnatal growth, reproduction and lactation through the regulation of metabolism and body composition, bone formation, neural stem cell activation, to erythropoiesis, myelopoiesis, thrombopoiesis and the inflammatory response. Dysregulated signalling from many of these receptors have been shown to play significant roles in promotion of a number of cancers [3–8]. These receptors primarily use JAK2 to activate the STAT regulators of gene transcription, but are also able to trigger the mitogen-activated protein kinase (MAPK) pathways, Akt/phosphoinositide 3-kinase (PI3K) and other pathways [9,10]. The textbook view of the activation process for JAK2 is that the 4-helix bundle cytokine possesses multiple binding sites for its receptor, and upon binding, these facilitate dimerization or multimerization of their cognate class 1 cytokine receptor. This then results in close apposition of the kinase domains of two membrane proximal receptor-associated JAK2s within the cell, and hence their transactivation. JAK2 activation is followed by tyrosine phosphorylation within the receptor cytoplasmic domain which generates docking sites for SH2 domain containing proteins such as STAT3 or STAT5, so that the docked protein is phosphorylated and activated. Receptor phosphorylation is accompanied by the direct JAK2 phosphorylation of other target proteins. This simple scheme has provided an explanation for the genomic and cytoskeletal changes induced by cytokine binding for over a decade.

## CLASS I CYTOKINE RECEPTORS

**Figure 1:**
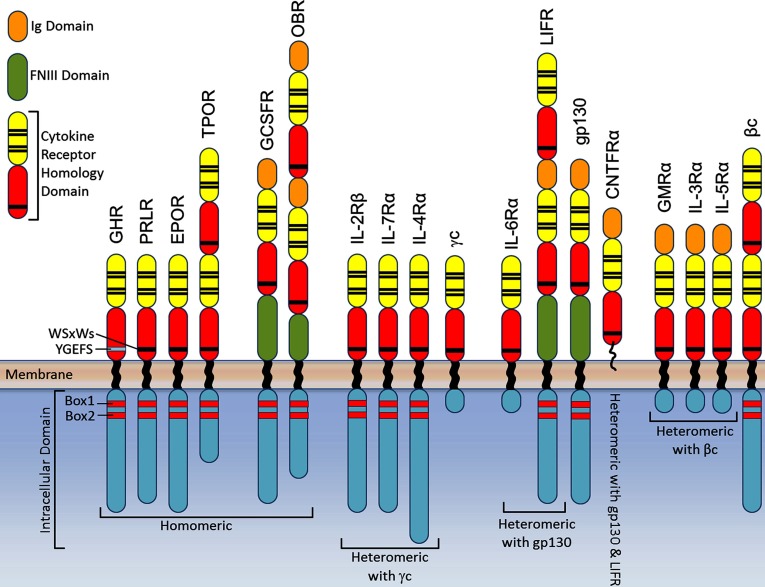
Domain structure of Class I cytokine receptors Domain structures depicted for the homomeric signalling receptors, and examples of heteromeric receptors that interact with the shared γc, gp130, βc and LIF-R (LIFR).

Before reviewing the receptor activation mechanism, a summary of class I cytokine receptor structure is necessary (Figure 1). As first shown for the GH receptor, these receptors are single pass transmembrane receptors with an extracellular domain (ECD) structure containing at least one cytokine receptor homology (CRH) domain which possesses two fibronectin III-like (FNIII) domains, each with an immunoglobulin-like β sandwich domain with seven strands in two layers [11]. The membrane proximal domain contains a WSxWS motif (YGeFS in the GH receptor) creating an aromatic stack which appears to be necessary for expression and stability [12]. Additional immunoglobulin or FNIII domains are present in many of these receptors [13], but the GH [14], prolactin [15] and EPO receptor [16] set of homomeric receptors, with just one CRH domain, is the simplest of these, and therefore most tractable for understanding the activation mechanism. In this case, the ligand/hormone has two asymmetrically placed binding sites, a high affinity site 1 and a lower affinity site 2 surface which are supplemented by receptor–receptor binding in the lower FNIII domain (site 3) upon binding of the ligand [17–20]. This is particularly evident in the GH receptor, and studies have shown that site 3 interaction is essential for signal transmission [21,22]. A second major feature of this receptor class is the proline rich Box1 motif, which together with the less-conserved distal Box2 sequence of aromatic and acidic residues serves to bind the JAK2 close to the inner cell membrane [23–27]. Recent NMR studies show that below this the cytoplasmic domains of the GH and prolactin receptors at least are essentially unstructured, with some transient alpha helix formation (unpublished data). This is concordant with the role of this domain in recruiting SH2 domain signalling proteins and the need for the relevant tyrosine residues to reach up to the JAK2 for phosphorylation.

A considerable amount is known about the structures of the ECDs of these receptors, beginning with the landmark crystal structure of the 2:1 complex of the archetypal dimeric GHR ECD with the GH [14]. Extracellular structures have subsequently been solved for several other members of the receptor family including the EPOR [16], PRLR [15], GM-CSFR [28], IL3Rα [29] IL-6R [30,31] and LIF-R [32,33]. Although all members of class I cytokine receptors have the characteristic two FNIII domain structure that is used to capture their ligand, there are distinct variations between similar groups of receptors within this class (Figure 1). As indicated, the simplest types have only two FNIII domains and are homodimers when bound to their activating ligand, whereas others require the interaction of two receptor subunits where one subunit is shared between different cytokine–receptor complexes [13]. These shared subunits, gp130 (also known as IL6ST, IL6β or CD130), LIF-R, and βc are the major signalling component of the receptor complex. Cytokines that utilize the gp130 signalling subunit include IL-6, IL-11, IL-27, LIF oncostatin-M (OSM), ciliary neurotropic factor (CNTF) and cardiotropin-1 (CT-1). Some gp130-cytokines, such as IL-6, homodimerize gp130 by first binding to their ligand specific α-receptor then to gp130 to form a signalling competent hexamer, whereas other gp130-cytokines, including LIF, CNTF, OSM and CT-1, heterodimerize gp130 with LIF-R to form signalling competent complexes [34]. The βc family of cytokines, which encompasses GM-CSF, IL-3 and IL-5, signal via heterodimerization of a major binding, cytokine-specific α chain and the shared βc [4]. Through the crystal structure of the GM-CSF extracellular receptor complex, it is thought that a dodecameric structure is formed which results in a signalling competent configuration where the signalling βc intracellular domains are in close proximity [28]. In general, it is believed that these receptors are activated by ligand-dependent receptor association.

## MECHANISM OF GH RECEPTOR ACTIVATION

**Figure 2:**
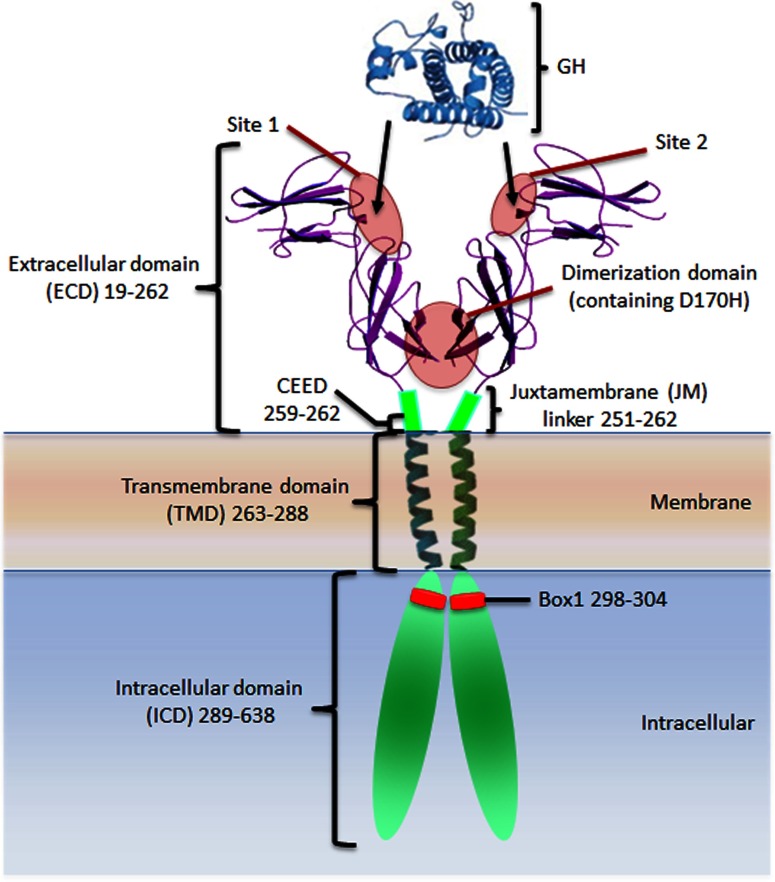
Growth hormone and the growth hormone receptor Crystal structure of the growth hormone and the growth hormone receptor extracellular domain with illustration representing the extracellular juxtamembrane linker, transmembrane domain, intracellular domain and Box1 motif. The growth hormone high affinity site 1 and lower affinity site 2 which are supplemented by receptor–receptor binding in the lower FNIII dimerization domain (site 3) are indicated.

The ligand-induced receptor association model stemmed from key publications by Genentech researchers who showed that the ECD of the GH receptor could be dimerized by GH both in solution [17,35] and in a crystal complex with GH [14]. Such dimerization was proposed to induce proximity of the associated JAK2s, hence their *trans*-activation and signal initiation. This model was supported by studies showing that 3 of 3 monoclonal antibodies (MAbs) to the ECD of the GH receptor acted as agonists, but only when bivalent [36], and by the bell-shaped dose–response curve which was explained by an inability of occupied receptors to dimerize if all of the receptors were taken in hormone site 1 interactions. Together with data showing that hindering of binding to the second receptor through substituting a bulky residue at Gly-120 in GH Helix 3 (i.e. in site 2; see Figure 2) resulted in an effective antagonist, the evidence for hormone-induced dimerization for this archetypal cytokine receptor appeared definitive. However, the cell line used for both experiments expressed a hybrid receptor consisting of the ECD of the human GH receptor fused to the N-terminal FNIII domain of the G-CSF receptor, so that the signalling which was observed was that of the G-CSF receptor, with receptor dimer formation likely to be influenced by its additional FNIII domains.

### Dimerization not key to receptor activation

Our laboratory developed a panel of MAbs to the purified rabbit GH receptor, including hormone binding site-directed MAbs, which were instrumental in cloning of the GH receptor [37]. When these and other MAbs were tested for agonism against the full-length rabbit GH receptor expressed in pro-myeloid FDCP-1 cells, it was found that only one of 14 MAbs was a weak agonist, whereas eight were effective agonists against the hybrid GH receptor/G-CSF expressing line [38]. The receptor epitope for the weak agonist antibody was subsequently mapped, and shown not to bind within the hormone binding site, but adjacent to it [39]. Therefore, dimerization of the receptor ECDs alone was insufficient to trigger activation; a specific receptor alignment or conformational change was needed. This was concordant with the different subunit alignments in the crystal structures of the EPO receptor ECD complexed to agonist or antagonist cyclic peptides [19].

An important additional consideration became apparent when the mode of action of the pegylated G120R antagonist B2036 was investigated. It was shown that B2036 bound to a receptor dimer at the cell surface i.e. the dimer formed without needing site 2 binding, suggesting that the receptor was pre-dimerized [40]. Shortly after this fluorescence co-patching was used to show that the EPO receptor exists as a preformed dimer at the cell surface [41], and this was concordant with the demonstration that the EPO receptor transmembrane domain (TMD) alone was able to self-associate [42] in a model system (ToxR assay) which located it to the inner membrane of *Escherichia coli* [43]. Our laboratory was able to show the same result with the GH receptor TMD using this ToxR assay, and identified residues which increased or decreased binding by mutagenesis [44]. Interestingly, it was observed that the prolactin receptor (but not the leptin receptor) TMD associated strongly with the GH receptor TMD using this technology [45]. This finding which was subsequently explored by Frank's group who showed co-immunoprecipitation of these two receptors in breast cancer T47D and prostate cancer LNCaP cells, and the ability of human GH to signal through the resulting heterodimer because hGH can bind to both site 1 and site 2 of the prolactin receptor [46].

Clearly these results did not support the ligand-induced dimerization model. The question was then formulated: Is the ligand-independent GH receptor dimer formed through ECD interactions as proposed from the EPO receptor ECD crystal structure [47] or did it involve the leucine-zipper like TMD association proposed by Langosch's group [48] which would be consistent with the ToxR assays? Gent et al. [49] answered this question with co-immunoprecipitation data based on co-expression of differently tagged GH receptors. Their study demonstrated that the ECD could be removed with proteinase treatment, yet a receptor dimer still remained. Our laboratory verified the existence of a constitutive dimer by co-immunoprecipitation with dual tags, and showed that it was not influenced by removal of JAK2 binding (Box1 mutation) and that removing nearly 300 residues of the cytoplasmic domain did not affect the extent of receptor dimer. Nevertheless, an increase in dimer formation was observed when the membrane associated receptors were pretreated with GH [50], suggesting that there may be some ligand-dependent receptor dimer formation. In order to establish if this was the result of detergent solubilization of the receptor destabilizing the constitutive dimer (which would then be stabilized by GH binding), N-terminal FRET was used to study the membrane bound receptor *in situ* [50]. This approach showed that addition of GH was without effect on extent of dimer, and through a series of truncations, that the TMD and juxtamembrane (JM) sequence were responsible for constitutive dimer formation. BRET reporters placed at the C-terminus verified this conclusion.

The lack of effect of ligand binding on the FRET of GH receptors with N-terminal FRET reporters indicated that there was no major conformational change in the ECD on ligand binding. Nevertheless, to clearly distinguish between a ligand-induced change in main chain conformation and a ligand-induced realignment of receptors, the crystal structure of the unliganded human receptor ECD was determined at 2.7 Å [50]. This revealed no major change in main chain position, although a rotation of 7–9 degrees between upper and lower FNIII domains was evident, which is minor in comparison with the conformational changes observed with tyrosine kinase ECDs on ligand-induced activation.

### A new model of receptor activation

Thus, receptor subunit realignment within a pre-existing dimer appeared to be the best candidate for signal generation by the architypal GH receptor. This would be predicted to reposition the Box1 motifs to which the JAK2s were bound, and should be visualized by placing FRET reporters nearby. Given the difficulty of the ligand accessing all of the receptors in vesicular cell membrane preparations or in intact cells, our group elected to use a Jun zipper to clamp all receptors in particular alignments, allowing consequence of this on proliferative signalling to be observed, while monitoring the positioning of FRET reporters placed close to the Box1 motif in parrallel [44,51]. The use of Jun zippers was based on a previous finding that the receptor could be made constitutively active by placing a Jun zipper at the receptor interaction site (site 3) in the ECD [52], a stratagem which can drive the growth of transgenic zebrafish [53]. Leucine zippers have been used to constitutively activate other cytokine receptors such as the GMRα, βc [54] and the gp130 common subunit [55]. Initially, the relationship between distance of the zipper from the cell surface and cell proliferation was studied in stably infected Ba/F3 cells. Beginning with the 12 residue linker, as the zipper approached the cell surface with 4 residue deletions, proliferative signalling increased to a maximum when the zipper was located close to the membrane surface. Surprisingly, this correlated with a greater separation of the FRET reporters below the membrane (37 residues below Box1) i.e. *FRET decreased* as the zipper was moved closer to the membrane surface. This was contrary to all previous views on receptor activation by these cytokine receptors, which, as stated above, posit that activation is associated with increased proximity of the Box1 JAK binding motifs.

Because of previous studies showing rotational positioning of the TMD was important for EPO and GH receptor activation [50,56], the effect of relative rotation of the TMDs was examined in the context of the leucine zipper constructs by introducing alanine residues (hence approximately 100° rotations) just above the TMD with the zipper at the cell surface [44]. In agreement with an analogous approach using a dimeric coiled coil for both the EPOR and the TPOR [56,57], there were rotational positions which favour constitutive signalling, and those that did not. The FRET reporters showed that the most active rotational position again corresponded to increased separation of the Box1 motifs. To determine if this finding was a result of reorientation of the FRET fluorophores, a flexible linker was introduced between the receptor and the FRET reporters. The decrease in FRET signal with receptor activation was still evident, so there was indeed a separation of the submembrane sequences with receptor activation [44,51].

In the light of the finding that apposition of the N-termini of the TMDs of the GH receptor with a Jun zipper was sufficient to activate the receptor and that introduction of three alanine residues positioned the construct in the strongest signalling orientation, the role of the conserved EED sequence that is located in place of the three alanines in the wild-type receptor was considered. Did this negatively charged sequence act as a barrier to receptor activation which could be overcome by binding of the bivalent cytokine ligand? To resolve this, the EED sequence was introduced within the Jun zipper construct instead of the three alanines. This decreased the level of constitutive activation, suggesting that negative charge repulsion was indeed maintaining the receptor in the inactive form. Again the FRET ratio increased compared with the three alanine sequence within the Jun zipper complex. To determine if this acidic sequence was acting as a signalling ‘gate’, the EED sequence was mutated to KKR within the wild-type full-length receptor and Ba/F3 cells were co-transduced with the wild-type receptor together with the charge complementation KKR mutant. This resulted in growth factor-independent proliferation of the these Ba/F3 cells, whereas cells transduced with either the wild-type or KKR constructs alone did not signal constitutively and proliferate. By placing FRET reports below the Box1 it was observed that constitutive signalling from co-expression of wild-type and KKR mutant receptors again resulted in an increased distance of the Box1 sequences while wild-type or KKR mutant alone did not change their FRET ratio [44].

In the three cases described above where the upper JM linker to the TMD was manipulated (length of JM linker, TMD rotation and charge reversal), an inverse relation between proliferative signalling in Ba/F3 cells and proximity of the Box1 sequences was observed. In view of the clinical and experimental finding that mutations within the lower JM sequence of the homologous TPO receptor can confer constitutive activation [58,59], our group investigated if this were also the case for the GH receptor. Using the least active Jun zipper construct (with 12 residue linker), individual alanine substitutions within the conserved SKQQRIK cytoplasmic JM sequence revealed enhanced constitutive activity only on substitution of the first lysine residue (K289A) at the predicted transmembrane boundary. This mutation again resulted in a decrease in FRET by reporters placed below Box1 in the parallel construct [44]. Thus, in all four cases, the evidence indicated that receptor activation results in separation of the Box1 sequences. Was this the case for GH itself? The earlier BRET study with reporters placed close to the cytoplasmic side of the plasma membrane had shown no change on addition of GH [50], but these experiments were done with high expression level where most of the receptor was trapped within the cells in aggresomes and vesicles, and inaccessible to the hormone. To circumvent this, the level of transient expression was greatly reduced, to the point where most of the receptor was located at the plasma membrane as visualized by fluorescence microscopy. Using these cells, plasma membrane preparations were prepared rapidly after GH addition and the change in FRET was determined with the Box1 proximal reporters. A substantial decrease in FRET following addition of GH was evident, and two important controls confirmed its physiological relevance: the G120R antagonist did not reduce the FRET and indeed, could block the hGH induced decrease. Secondly, introducing a Laron dwarf mutation in Site 3 (D170H) of the ECD prevented the decrease in FRET induced by the hGH [44,51].

Recently, Frank's group published a study on the GH receptor activation process based on complementation of luciferase fragments which concluded that GH binding *increases* complementation i.e. brings the cytoplasmic domains closer together [60]. In this study, the fragments were placed either at the C-terminus of the receptor or below the Box1 motif in a similar position to our FRET reporters. In both cases, there was an increase in complementation on GH addition, which, given the unstructured nature of the cytoplasmic domain, would exclude change in rotational alignment as a reason for the increased proximity. There are two problems with this experimental approach. First, although it is suitable for detecting interacting proteins when the two enzyme fragments are in close enough proximity to associate to form a functional enzyme (e.g. dimerization), once associated the two parts will not easily detect separation due to their attractive atomic forces, particularly if fused to the long flexible cytoplasmic domain. Secondly, the extent of GH-induced complementation in this study varied 10-fold even with the same dual fragment expressing clonal cells, which is surprising. The baseline levels in these studies exceeded 80% of the total signal in several cases, which would be consistent with a small population of monomer (15–20%) associating in response to hormone binding, while the major part of the receptor remained as the constitutive dimer. The existence of some monomer would also be consistent with both the full length (C-terminal tag) and membrane proximal tags giving a similar modest increase in luciferase activity with GH addition.

Given an increased separation of the Box1 motifs with receptor activation, it is necessary to understand the TMD helix movements which could induce such a separation in response to GH-induced apposition of the upper JM sequences. Since no NMR or crystal structure of interacting TMDs was available for these class I receptors it became necessary to use molecular dynamics modelling to investigate the likely structure of the two GH receptor TMDs. This required knowledge of the helix alignment in at least one state. Therefore, every residue in the TMD and upper JM domains were converted individually to cysteine, then the crosslinkers MTS-2-MTS or Cu-o-phenanthroline were added to the cells or cell membranes, and these were subjected to SDS gel electrophoresis in non-reducing gels. This revealed the basal state helix orientation in the absence of ligand, evident when the crosslinked residues were plotted on a helix wheel projection. One side of the helix gave strong crosslinking, which included Phe-273, Phe-276, Val-280 and (weakly) Phe-283 at the limit of crosslinker penetration. Although it was not possible to show a difference in this pattern on addition of hormone (likely a result of aggresomes/vesicle localization), it was possible to show in intact cells that Asp-262, within the EED sequence, formed a crosslink more strongly in the presence of GH [44]. This was consistent with the notion that ligand-induced proximity of the upper JM sequence is essential for receptor activation. This notion was strengthened by the finding that some spontaneous formation of disulfide bonds occurred during biosynthesis which resulted in a variable level of STAT5 tyrosine phosphorylation. On systematic analysis of this, it became evident that crosslinking from Ser-255 in the upper JM linker to Leu-269 at the N-terminus of the TMD was able to induce spontaneous receptor activation in the transient transfection system [44]. This substantiated the finding with the Jun zipper that closure of the upper TM helices triggers receptor activation.

**Figure 3:**
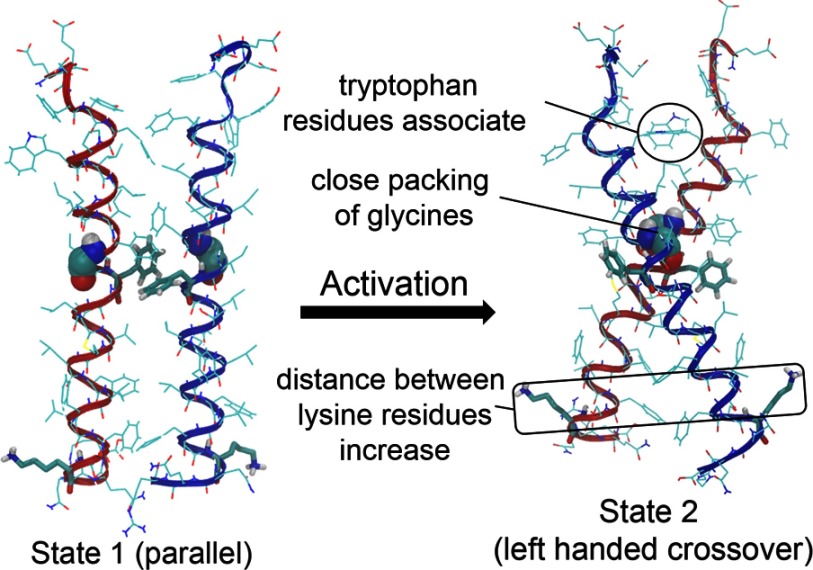
Transition of the GH receptor TMDs The interaction of the GH receptor TMDs in the inactive parallel State 1 and active crossover State 2 are shown.

Our group sought to understand these findings through molecular dynamics modelling, first *in vacuo*, then in DPPC membrane bilayers [44]. Using coarse grain models to model the approach of two GH receptor TMD helices in DPPC, it was observed that the helices first converge at their C-termini when their centres of mass are approximately 20 Å apart. The main reason for this is repulsive interactions at the N-termini. As the helices approach more closely, aromatic Phe-276 and Phe-283 interact, and together with Thr–Thr hydrogen bonding, this stabilizes a parallel form with a helix interaction face evident in the cysteine crosslink pattern. Further approach, as would be induced by ligand binding, resulted in a rotation of helices and separation of their C-termini, so that the two glycines of the conserved GxxG motif packed together. This meant the phenylalanines were rotated out of contact, resulting in a left-handed crossover dimer with lowest PMF (potential of mean force) (Figure 3). The PMF of the parallel form (State 1) is close to that of the crossover form (State 2) so that the transition between the two forms would be relatively facile. Importantly, the predicted free energy of binding for the two helices (approximately 10 kcal/mol) indicated little monomer will be present. The latter prediction was borne out by homo-FRET efficiency measurements at the cell surface using confocal microscopy which showed that little monomer was detectible at moderate expression levels. It was gratifying that it was possible to recapitulate State 2 *in silico* by introducing disulfide bonds at Glu-260 and Ile-270 in the JM and upper TMD helix locations because this mutant had substantial constitutive activity experimentally [44].

Atomistic modelling based on the coarse grain models was then applied to estimate sulfur–sulfur distances to match the crosslink data, and this agreed with the experimental results. The atomistic modelling also highlighted the role of Trp-267 at the upper TMD helix boundary, which rotates away from the centre in State 2 to facilitate upper helix apposition. Lys-289, which anchors the lower C-termini at the water-membrane boundary in State 1, is in an unfavourable position opposing helix interaction in State 2 [44]. This provided an explanation for the finding that alanine substitution of only this JM residue resulted in constitutive activity.

Finally, with only minor residue insertions, the Genentech 2:1 ECD complex docked easily with State 2 [44], and when the GH was removed *in silico*, a rotation of the ECDs occurred. This had been previously observed by Poger and Mark [61] with the 2:1 complex *in silico*. Importantly, docking of the 2:1 complex with the two TMDs in State 2 resulted in close proximity of Cys-259s, which are known to form a disulfide bond on binding of GH [62].

## THE MECHANISM OF JAK2 ACTIVATION

**Figure 4:**
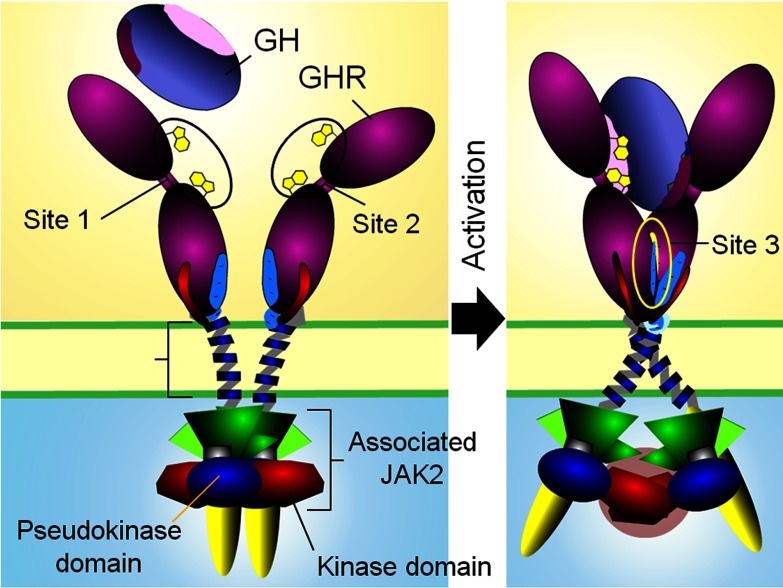
Activation model of homodimeric class I cytokine receptors shown for the archetypal GH receptor

The model derived from these experimental and *in silico* findings predicts that binding of a bivalent ligand to the GH receptor will bring the ECD site 3 structures into proximity and then association through subunit rotation, and this will facilitate closer apposition of the upper JM sequence by overcoming the electrostatic repulsion of the JM EED sequence. Apposition will be stabilized with the aid of hydrogen bonds between Glu-260 of receptor 1 and the amide of Trp-267 in receptor 2, and between the backbone carbonyl of Phe-257 and amide of Glu-260 in receptor 2 [44]. Apposition of the upper TMD will result in relative rotation of the helices from the parallel State 1 to the left hand crossover State 2, with widening of the distance between the C-termini of the TMD helices. The leads to the key question: how could lower TMD separation result in activation of the JAK2 tyrosine kinases associated with the membrane proximal receptor Box1 motifs? Of particular relevance here is the existence of a pseudokinase (PK) domain adjacent to the kinase domain, which is known to be inhibitory [63]. Interestingly, of the 518 protein kinases, 48 contain PK domains, but only five of those contain an additional kinase domain. These are the four JAKs and the serine/threonine kinase GCN2 [64]. Hence any model for activation must remove the PK domain away from the kinase domain, and it is notable that myeloproliferative neoplasms with constitutive JAK2 activation possess point mutations in the PK domain or the linker to the adjacent SH2 domain, rather than the kinase domain. Since Lodish's group has shown that the constitutively active V617F mutant of JAK2 does not manifest its activity unless the EPO receptor is present [65,66] (or if the mutant is overexpressed [67]), the activation process is likely a *trans*-mediated activation event within a receptor/JAK2 dimer rather than a *cis*-activation process. If the PK inhibitory domain of one JAK2 was blocking the kinase domain of the other JAK2, then pulling the complex apart (such as would occur with separation of the Box1 motifs) would remove this *trans*-inhibition, and place the two kinase domains into proximity which could induce *trans*-activation (Figure 4 and animation at http://web-services.imb.uq.edu.au/waters/hgh.html). Given the nature of this process, supporting evidence for this model would need to be obtained *in vivo*.

**Figure 5:**
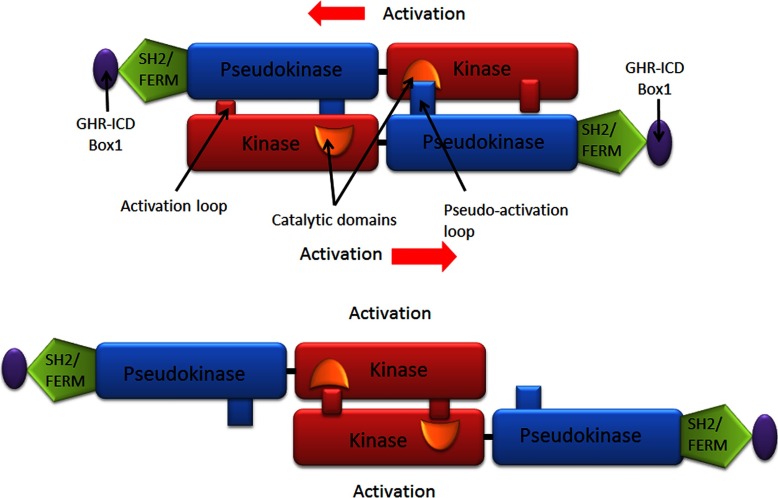
Model of JAK2 activation for the GH receptor In the inactive state (top), two JAK2 molecules, each bound to a GH receptor intracellular domain, interact so that the kinase domain of one JAK2 is inhibited by the pseudokinase domain of the other JAK2. GH binding causes separation of the ICD Box1 motifs and leads to separation of the pseudokinase–kinase *trans*-interaction and results in kinase–kinase *trans*-interaction and activation (bottom).

Our group sought *in vivo* support for the JAK2 *trans*-inhibition model initially by introducing FRET reporters in place of the PK domain or at the C-terminus, where the kinase domain is located. The prediction was that receptor activation would result in a decrease in FRET for the PK domain located reporter, but an increase for the kinase domain located reporter. Using the wild-type GH receptor and the EED to KKR charge reversal receptor construct either separately or together, it was possible to ensure that essentially all of the receptors would be in inactive State 1 (when wild-type or KKR mutant were expressed alone) or consist of a subset of active State 2 receptors (when co-expressed). A decrease in FRET for the PK position reporter was indeed observed when transiently transfected HEK293T cells expressed JAK2 FRET reporters with both wild-type and KKR mutant receptors together (giving constitutive activation) compared with cells with expression of a single receptor construct alone. Conversely, similar analysis using the kinase position reporter showed an *increase* in FRET demonstrating that activation results in an increase in distance between the JAK2 PK domains while bringing the kinase domains in closer proximity, as predicted. Further *in vivo* evidence for the *trans*-model was obtained by reversing the positions of the kinase and PK domains in the JAK2 expression construct, then co-transfecting this construct with a construct for wild-type JAK2 (along with the GH receptor and STAT5) into JAK-deficient γ2A fibrosarcoma cells. The JAK2 *trans*-model (Figure 5) predicts constitutive activity with this co-transfection, since the combination would remove the PK domain from the other kinase domain and appose the kinase domains. This was indeed evident in multiple experiments, but only in the presence of the receptor, consistent with the *trans*-activation mechanism [44].

Further *in vitro* studies were undertaken to strengthen the evidence for this model (Figure 5). It was shown that fluorescently tagged JAK2 PK–kinase pairs can associate with other such pairs by Alpha screen, and in addition, single molecule brightness coincidence data consistent with dimerization of PK–kinase pairs under cell free conditions was obtained [44]. Finally, molecular dynamics were used to dock the crystal structures of the kinase and PK domains with HADDOCK, and that one such pair could dock *in trans* with another such pair in an orientation consistent with the sliding apart model [44]. Consistent with this arrangement, the recent publication describing the solution structure of PK-K of JAK2 by SAXS shows an elongated structure congruent with this *in trans* model [68]. Moreover, activating mutations within the PK domain of JAK2 result in activation of the kinase activity of JAK1 and TYK2 in a heterodimer, supporting such a *trans* arrangement [69].

This *trans* view of the JAK activation process contrasts with previous proposals which posit *cis* autoinhibition with displacement of the PK inhibitory module by some sort of steric interaction resulting from closer apposition of the Box1 motifs and associated FERM domains (see review [70]). Clearly this view is not congruent with the evidence supporting separation of the Box1 motifs upon receptor activation. Available crystallographic data for TYK2 indicates a *cis* arrangement (although the interdomain linker is not resolved in the structure) [71], as does recent molecular dynamics modelling of JAK2 supported by limited charge reversal mutagenesis [72]. One would expect such an arrangement with structures deriving from the docking of isolated kinase and PK domain crystal structures [73,74]. However, as was described in Brooks et al. [44], it is possible to dock the PK–kinase domain pairs *in trans* using the binding surfaces proposed by earlier studies which locate the key V617F PK mutation close to the activation loop of the kinase domain [75–77]. Our group has also been able to dock the crystal structures of the kinase and PK domains at the surfaces recently identified by Shan et al. [72] in a *trans* configuration (unpublished data), so these new structures do not exclude such a *trans* configuration. It is only by *in vivo* experiments using such techniques as FRET with the receptor dimer and intact JAK2 that this issue could be resolved. Electron microscope image reconstruction appears to lack the resolution needed for resolving this problem [78].

### Therapeutic Inhibition of JAK2

Inhibition of JAK2 is an important clinical target, particularly for myeloproliferative disorders. These disorders commonly result from constitutive activation of JAK2 signalling through mutation of JAK2, frequently via the JAK2 V617F mutation (especially in polycythaemia vera), but also from JAK2 exon 12 mutations, or mutation of the TPO receptor (MPL), or mutations of LNK, a negative regulator of JAK2 [79–81]. There has been considerable effort put into the development of therapeutically effective inhibitors of JAK2; however, to date these have had limited success. There are currently a little over 10 JAK selective inhibitors in clinical trials, however few are specific only for JAK2 with the exception of gandotinib and BMS-911543. Only ruxolitinib (selective inhibitor of JAK1 and JAK2) has received Food and Drug Administration (FDA) approval for the treatment of myelofibrosis, while many other compounds have been discontinued due to toxicity [80,82]. Ruxolitinib does improve constitutional symptoms and splenomegaly, but does not substantially reduce mutant allele burden in patients and these benefits come at the cost of frequent anaemia and thrombocytopenia [83,84]. Pacritinib, a dual JAK2 and FLT3 inhibitor has showed significant promise in early phase trials with limited haematologic toxicity and is currently in Phase III clinical trials for myeloproliferative disorders [85,86]. Clinical trials of polycythemia vera and essential thrombocythemia patients with lestaurtinib, a multikinase inhibitor of JAK1, JAK2, JAK3, FLT3 and TRKA/B/C has shown to modestly reduce JAK2-V617F allele burden and spleen size [80,87]. Tofacitinib (tasocitinib), a pan-JAK inhibitor has been FDA approved for rheumatoid arthritis and is being studied for the treatment of a range of disorders including polycythemia vera, psoriasis, ankylosing spondylitis and for the prevention of transplant rejection [82,88,89]. Further improvements to the mechanisms of JAK2 inhibition and increased specificity should lead to more effective therapeutic outcomes.

## RELATIONSHIP OF THE GH RECEPTOR ACTIVATION MODEL TO OTHER CLASS I CYTOKINE RECEPTORS

Several members of the class I cytokine receptor family have been reported to exist as constitutive inactive dimers, notably PRLR [90], EPOR [42], gp130 and gp130/LIF-R [91,92], implying that activation must result from structural changes within the receptor transmitted through the TMDs. Studies on gp130 also suggest that cytokine binding brings the C-terminal membrane proximal ECD into close proximity [78,93]. Since no high-resolution structure for the TMD and membrane proximal regions has been solved for any class I cytokine receptor, our group has begun to investigate the possibility of a unified mechanism using receptor chimeras. In preliminary studies, it has been found that fusing the EPOR ECD and TMD to the ICD of GHR results in a receptor that signals in response to the EMP-1 agonist peptide, but not the EMP-33 antagonist (unpublished results). Similar studies from other groups have been performed with receptor chimaeras between EPOR and gp130 [94], as well as between GHR and G-CSF-R [95]. Given the nature of the helix dimer transition from a parallel form to a crossover form as a result of N-terminal apposition and helix rotation, it is likely that any helices which can pack together in this way will be able to signal. This accounts for the finding of Frank's group that the GH receptor TMD helix can be replaced by the LDL receptor TMD helix, and still signal effectively [96]. It is notable that all class I receptors examined have acidic residues proximal to the outer membrane, providing the possibility of a gating mechanism similar to the one we have described for the GH receptor. Although these studies are suggestive of a common mechanism, this hypothesis is yet to be confirmed.

Activating mutations in the TMD of class I cytokine receptors have been reported for the G-CSF receptor where they have been found to induce chronic neutrophilic leukaemia (T617N and T617I, T595I and T592A) [97–99]. Similarly, the S505N and S505A mutations in *mpl*, the TPO receptor results in familial essential thrombopoiesis [100]. In both cases, it was initially thought that the mutation would facilitate receptor dimerization in the absence of ligand (e.g. additional hydrogen bonding for T617N and for S505N). However, the identification of further mutations which do not possess this ability (e.g. T617I and S505A) argued against this. In the light of evidence for constitutive dimerization of the TMD helices, the constitutive activity could be the result of rearrangement of the helix packing analogous to our State 1-State 2 transition. In support of this, Lee et al. [101] undertook comprehensive molecular dynamics modelling of the S505 mutations in TMD of *mpl*, and concluded that ‘mutations within the TMD region could result in conformational changes including tilt and rotation (azimuthal) angles along the membrane axis…which may alter the conformation of the adjacent flexible intracellular domain’. They reached a similar conclusion for W515 mutations which, as mentioned, parallels the inhibitory K289 residue that sits at the lower TMD boundary of the GH receptor. Similarly, the data of Ding et al. [100] showing increased dimer level in the S505N *mpl* mutant lacking an ECD would be in this case explained by the short DSG linker (7.7 Å) crosslinking the amino-terminal residues more effectively if the mutation had altered helix packing to the State 2 orientation. Further support is provided by Matthews et al. [102] who report that the TPO receptor TMDs self-associate, and that cysteine crosslinking revealed a TMD interaction interface, whereas the S505N mutation promotes the formation of a different dimerization interface, implying that activation involves a subunit rotation.

## SIGNALLING BIAS

This review would be incomplete without considering the issue of signalling bias by these receptors. There are several reports that altering the receptor subunit alignment results in alternate signalling patterns. For example, Liu et al. [103] studied EPO receptor signalling with an HA-tagged anti-fluorescein ScFv attached to the N-terminus, then triggered receptor activation with a variety of ligands including EPO itself and a fluorescein dimer. They also introduced 1–4 alanines at the lower membrane boundary to rotate the subunits in 100° increments. Marked differences were seen between STAT5 and ERK2 activation ability with particular rotations when either EPO or fluorescein dimer were applied. When the ScFv was fused directly to cytokine receptor domain 2, these differences became even more apparent. Likewise, Seubert et al. [56] found positional differences in constitutive activation between STAT5 and ERK when they rotated the EPO receptor signalling unit with the aid of a coiled coil replacing the ECD. A similar finding was reported by Staerk et al. [57] for the TPO receptor using the coiled coil rotation method within the constitutive dimer.

Our group became aware of biased signalling when the role of the F’G’ loop in the lower cytokine receptor domain was studied [104]. This loop differs in its conformation between the crystal structure of the hormone bound form and the G120R antagonist bound by forming a strong salt bridge with the upper domain via Arg-39 in the presence of hGH. It was surmised that this loop rearrangement could relate to the receptor activation process. Therefore, the loop was mutated so that its conformation matched the antagonist form, and its signalling ability was studied in promyeloid FDC-P1 stable clones matched for expression. It was observed that GH-induced proliferative signalling was strikingly reduced, whereas ability to activate JAK2 and STAT5 was not altered. However, ability to activate ERK was strikingly reduced. When this loop mutant was studied after stable expression in Ba/F3 cells, JAK2/STAT5 activation ability was normal, but again ERK activation was again reduced. To investigate the role of the TMD orientation in this apparently biased signalling, helix disruptive Gly-Pro pairs were introduced into the TMD helix in different locations and STAT5 and ERK signalling in response to GH were ascertained with reporter assay. There were very evident differences in the ratio of these two with different TMD helix configurations. It was then determined if ERK activation could be seen in the absence of JAK2 activation by disabling the Box1 sequence with four alanine substitutions. Indeed, ERK activation in response to GH was still apparent, even in γ2A cells which do not express JAK2. The group went on to make targeted knockin mice with the Box1 sequence disabled, and found that bovine GH administration could still activate hepatic ERK signalling in these knockin mice. How was the receptor activating ERK? It was shown that ERK activation was via a Src family kinase (*lyn*) which associated with the receptor below the Box1 sequence, and that phospholipase Cγ was utilized by *lyn* to activate ERK signalling. It is notable that others have shown that JAK2 activates ERK from the GH receptor in cell lines [105] so it was concluded that the relative amount of SFK and JAK2 would be a determinant in choice of signalling pathway.

Thus the biased signalling was not a consequence of altered JAK2 catalytic mechanisms, but rather a result of utilizing an alternate tyrosine kinase. This may well be the case for other class I receptors which have been shown to associate with SFKs such as the prolactin receptor [106,107], EPO receptor [108,109], IL-6 receptor [110], GM-SCF receptor [111–113], the TPO receptor and others [114].

## CONCLUDING REMARKS

Activation of class I cytokine receptors by ligand binding requires the activation signal to be transmitted to their associated intracellular JAKs by structural rearrangements of the ECD linker and TMD. By unravelling the activation mechanism for these receptors, valuable insights for novel approaches in developing clinically relevant drugs will be gained. The critical nature of the ECD linker and TMDs is highlighted by a number of clinical and experimentally introduced activating mutations for a number of class I cytokine receptors including TPOR (S505N) [100], IL-27R (F523C) [115], βc (V449E) [113], and a number of mutations for EPOR [116] and GHR [44,51]. These studies expose this vital region as a potential Achilles’ heel for this class of cytokine receptors. Therefore, molecules that bind to the ECD linker and/or TMDs are potential modifiers of signalling, either by inhibiting activation or activating the receptor in the absence of ligand. Such molecules have already been obtained by high-throughput screening for TPOR including eltrombopag (the US FDA approved PROMACTA) which activates TPOR by binding to N-terminal residues of the TMD and ECD linker sequence for the treatment of thrombocytopenia. These molecules have the major advantage of activating a receptor that is insensitive to ligand due to mutations in ligand binding residues. For example, the F104S mutation in TPOR does not respond to TPO, but does respond to LGD-4665 which interacts with the TMD [117]. Similarly, short transmembrane proteins (‘traptamers’) have been developed that specifically activate EPOR [118]. Based on an understanding of the activating movements of class I cytokine receptors as proposed in the present study, it should be possible to progress to receptor-specific small agonists and antagonists of therapeutic value for otherwise intractable disorders.
